# A Memoir on the Pathology and Treatment of Leucorrhœa, Based upon the Microscopical Anatomy of the Os and Cervix Uteri

**Published:** 1852-11

**Authors:** 


### ECLECTIC DEPARTMENT,

### AND SPIRIT OF THE MEDICAL PERIODICAL PRESS.

---

*A Memoir on the Pathology and Treatment of Leucorrhœa, based upon the Microscopical Anatomy of the Os and Cervix Uteri.* Read before the Royal Medical and Chirurgical Society. By W. Tyler Smith, M. D., Physician-Accoucheur to St. Mary's Hospital.

The author first directed attention to the minute anatomy of the os and cervix uteri; and here, at the outset, he was desirous of expressing his warmest thanks and obligations to Dr. Arthur Hassall, for his valuable assistance in the microscopical part of the investigation, and without which he could not successfully have prosecuted his researches. The mucous membrane of the os and cervix uteri, like the mucous membrane of other parts, consisted of epithelium, primary or basement membrane, and fibrous tissue, blood vessels and nerves. But as there were some special characteristics pertaining to this tissue, he proposed, for the convenience of description, to examine, first, the mucous membrane of the os uteri and external portion of the cervix; and secondly, the mucous lining of the cervical cavity or canal. The epithelial layer of the former of these situations was tesselated or squamous, and so arranged as to form a membrane of some thickness; by maceration, it could be easily detached, and it was then found closely to resemble the epithelial covering of the vagina, with which it was continuous. Beneath this epithelial layer was the basement membrane covering numerous villi or papillæ, which studded the whole surface. Each villus contained a looped blood-vessel, which, passing to the end of the villus, returned to its base, and inosculated with other blood-vessels of the contiguous villi. These villi had been mistaken for mucous follicles, usually described as covering the surface of the os uteri; but the microscope failed to discover any distinct follicular structure in this situation. When a thin section of the surface of the os uteri was examined by a low power, the points of the villi could be seen as dark spots through the epithelial layer. A careful examination exhibited these spots as slightly depressed in the centre, yet nevertheless slightly elevated in their margins, nipple-shaped, and containing red points, which were the terminations of the looped blood-vessels. These appearances were produced by the villi being obscured by their epithelial covering. The thick layer of scaly epithelium, and the villi with their looped vessels, were the principal anatomical features of the mucous membrane of the os and external part of the cervix uteri; and these structures played an important part in the pathological changes which occurred in the lower segment of the uterus in leucorrhœa. Between the margin of the lips of the os uteri and the commencement of the penniform rugæ, within the precincts of the cervical canal, a small tract of smooth surface was usually found, which to the naked eye seemed of more delicate structure than the neighboring parts, and when examined by the microscope was found to be composed of cylinder epithelium, arranged after the manner of the epithelium covering the villi of the intestinal canal. The cylinder epithelium covered in this situation villi two or three times larger than the villi upon the surface of the os uteri—so large, indeed, as to be visible to the naked eye when viewed by transmitted light. Within the cavity of the cervix uteri, the mucous membrane contained four columns of rugæ, or folds, arranged in an oblique, curved, or transverse direction; and between these columns were four longitudinal grooves. The two sulci in the median line, anteriorly and posteriorly, were the most distinct; and of these, the sulcus of the posterior columns was the most strongly marked. In the normal state, the transverse rugæ, with the fossæ between them, were filled with viscid, semi-transparent mucus; and when this was brushed away, a reticulated appearance, caused by numerous secondary rugæ, was visible. The author gave a very minute description of these four rugous columns, and the furrows between them, which was illustrated by some very beautiful drawings of the cervical canal, displaying the rugous columns and fossæ of the natural size, and magnified nine and eighteen diameters. The latter power showed a large number of mucous fossæ and follicles, crowding the depressions between the rugæ and the rugous elevations also. The author mentioned that a healthy virgin cervix, of normal size, contained at least ten thousaud mucous follicles. This anatomical arrangement secured a vast extent of superficial surface, which was still further increased by the presence of villi similar to those found in the lower part of the cervix; they were found in considerable numbers on the large rugæ and other parts of the mucous membrane in this situation. By this disposal of the mucous membrane of the canal of the cervix, a very large extent of glandular surface was obtained for the purpose of secretion. In effect, the cervix was an open gland; and in the opinion of the author, it was in this part of the utero-vaginal tract that the principal seat of leucorrhœa would be found to exist. There was an analogy here which should not be lost sight of, bearing, as it did, on the pathology and treatment of leucorrhœa, which was, the similarity which existed between the skin and the mucous membrane of the vagina and the external part of the os and cervix uteri. The resemblance, in these situations, was certainly much nearer to the cutaneous structure than to the mucous membrane of more internal parts. These analogies were strongly confirmed by what was observed of the pathological conditions to which these parts were liable, and by the effect of therapeutical applications. The author dwelt on the fact that the epithelium of the os uteri and external portion of the cervix was constantly squamous, and that the epithelium immediately within the os uteri was cylindrical but not ciliated, but that in the rugous portion of the cervical canal the cylindrical epithelium became ciliated. The mucus secreted by the glandular portion of the cervix was alkaline, viscid, and transparent; it adhered to the crypts and rugæ, so as to fill the canal of the cervix. It consisted chiefly of mucous-corpuscules, oil-globules, and occasionally dentated epithelium, all entangled in a thick, tenacious plasma; it was remarkable for its tenacity; while the mucus found in the lowest part of the canal was thinner in appearance. There was an essential chemical difference between the vaginal mucus and the secretion of the interior of the canal of the cervix; the first was always acid, and the latter invariably alkaline. Mr. Whitehead, of Manchester, had noticed this fact, and the observations of the author confirmed his views. The acid of the vaginal secretion was more than sufficient to neutralize the alkaline secretion of the cervix, and when any secretion from the cervical canal entered the vagina it became curdled from the coagulation of its albumen. It was worthy of note, that that part of the mucous membrane of the uterus and vagina which resembled the skin was the only part which, like the skin, furnished an acid secretion. The vaginal mucus was a simple lubricatory fluid. But the uterine cervical mucus had other uses besides that of lubrication; in the healthy condition, in the intervals of the catamenia, it blocked up the passage from the vagina to the fundus; it thus defended the cavity of the uterus from external agencies, and from its alkaline character afforded a suitable medium for the passage of spermatoxoa into the uterus. Having stated his views of the structure of the utero-vaginal mucous membrane, the author expressed his opinion that the glandular portion of the cervix uteri was the chief source of the discharge in leucorrhœa. Leucorrhœa, in its most simple and uncomplicated form, was the result of an increased activity of the glandular portion of the cervix. A follicular organ, which should only take an active condition at certain intervals, became constantly engaged in secretion. Instead of the discharge of the plug of mucus at the catamenial period, an incessant discharge was set up. At first the discharge was but an unusual quantity of the elements of the healthy mucus of the cervix. The quantity increases, and becomes a serious drain to the constitution, and the glandular cervix in some cases becomes so excitable, that any unusual stimulus, even mental emotions, provokes an augmentation. The author next referred to the conditions under which the epithelium of the os and external part of the cervix uteri and upper portion of the vagina might be partially or entirely removed. The mucous membrane then presented an intensely red color, from the presence of the naked villi, and an appearance of roughness or excoriation presented itself. He thought that among the causes which produced this aspect of ulceration, were eruptive disorders, similar to herpes or eczema, which strongly marked the analogy between this tract of mucous surface and the skin. He had observed cases in which an occasional herpetic eruption upon the os uteri always produced herpes præputialis in the husband. But the most frequent cause of denudation arose from the alkaline mucus discharge of the cervix irritating the acid surface of the os uteri, and causing the rapid shedding of the epithelium round the margin of the os. A microscopical examination was given of the various discharges met with in these affections, in making which the author was assisted by Dr. Handfield Jones and Dr. Hassall. In cervical leucorrhœa the discharge consisted of quantities of mucous-corpuscles, and in severe cases pus-corpuscles and blood-discs, with fatty matter, involved in a transparent plasma. The epithelial debris is constantly present, but in limited quantity. In vaginal lucorrhœa, including the secretions of the external portion of the os and cervix uteri, the plasma is opaque, and contains myriads of epithelial particles in all stages of development, with pus and blood globules when the villi are affected. When a circumscribed ulcer upon the os uteri is visible to the naked eye, after death, such as occurs in eruptive affections of the os and cervix, and is examined by the microscope, with a low power, it is found to consist of a base from which the villi are entirely removed, or in which only the scattered debris of villi remain; and surrounding this base there is a fringe of enlarged villi, partially or entirely denuded of epithelium. The character of the so-called ulceration of the os uteri was detailed, and the nature of the discharges described. The author then observed that if any division of leucorrhœa were made, two principal forms must be recognized:

I. The *mucous* variety, secreted by the follicular canal of the cervix.

II. The *epithelial* variety, in which the discharge was vaginal.

With respect to the so-called ulcerations of the os and cervix, two kinds of morbid change would be observed:

I. *Epithelial abrasion*, by far the most common, in which the epithelium alone was deficient.

II. *Villous abrasion*, *erosion*, or *ulceration*, in which the villi are affected by superficial ulceration.

It was to the villi, denuded of epithelium and partly eroded, that the marked forms of granular os uteri were owing. The ovules of Naboth, often referred to by writers as obstructed follicles, the author had found to be in reality an eruptive disease of the mucous membrane analogous to a cutaneous affection. In these affections of the cervix uteri it frequently happened that the cervix uteri was partially everted, and the deep-red surface covered by vascular villi thus exposed, had frequently been mistaken for breach of continuity in the mucous surface. The author then offered some remarks on the practical deductions which might be drawn from the present investigation. The glandular structure of the parts from whence the leucorrhœal discharge arose, pointed to the influence of constitutional causes, and exemplified why this affection should be so common in women of strumous habit and leuco-phlegmatic temperament; it vindicated the importance of constitutional treatment, and directed attention to the more rational employment of topical remedies; and it was evident that the profuse application of caustics, as recommended by the French school of uterine pathology, was both unnecessary and unscientific. He admitted that leucorrhœa of the cervical canal was sometimes cured by the use of caustics to the os uteri, but in these cases they acted as counter-irritants to the glandular structure. The indications of treatment, based on a knowledge of the minute anatomy of the os and cervix uteri, and the study of its pathology in leucorrhœa, appeared to the author to require constitutional medicines and regimen, with local applications. Local measures, to be of any use in cervical leucorrhœa, should be applied, not to the vagina, nor the os uteri, but to the canal of the cervix. In vaginal or epithelial leucorrhœa, common injections were serviceable; but in cervical or mucous leucorrhœa no benefit could result unless the injection passed into the cervix. He mentioned the methods he adopted to secure this result, and concluded by expressing a hope that the prosecution of these researches might prove serviceable, by rendering a troublesome class of maladies more intelligible than they had hitherto been, and by tending to correct errors of practice, and to indicate the just value of constitutional and topical remedies.

[Dr. Tyler Smith's Paper was illustrated by a number of beautiful drawings, which excited great attention among the Fellows, representing the novel points described in the paper, and which were made under the superintendence of Dr. Hassall.]

At the conclusion of Dr. Smith's paper, the President observed that he should be happy to hear any observations upon it from the Fellows. After a short pause,

Dr. Locock rose and said that he regretted an appointment obliged him to leave the society immediately, but he could not do so without first offering to Dr. Tyler Smith, for his very admirable paper. He could scarcely remember an occasion on which he had listened to a paper with greater interest, or from which he had derived so much instruction. The present communication was, in his opinion, a step in the right direction, and he felt convinced that researches of this kind would eventually lead to a better understanding and an improved treatment of what was most certainly a very intractable class of disorders. He was glad to learn the author intended to pursue the subject, and he should certainly look forward with great interest to the progress of his further investigations. (Cheers.)

